# Unraveling the neuroprotective effect of perampanel and lacosamide combination in the corneal kindling model for epilepsy in mice

**DOI:** 10.1002/ame2.12524

**Published:** 2025-01-23

**Authors:** Saba Tehreem, Azka Sabir, Maryam Farooq, Waseem Ashraf, Faleh Alqahtani, Tanveer Ahmad, Imran Imran

**Affiliations:** ^1^ Department of Pharmacology, Faculty of Pharmacy Bahauddin Zakariya University Multan Pakistan; ^2^ Department of Pharmacology and Toxicology, College of Pharmacy King Saud University Riyadh Saudi Arabia; ^3^ Institut pour l'Avancée des Biosciences, Centre de Recherche UGA/INSERM U1209/CNRS 5309 Université Grenoble Alpes Grenoble France

**Keywords:** behavioral studies, corneal kindling, lacosamide, learned helplessness, oxidative stress, perampanel

## Abstract

**Background:**

Scientific evidence to guide clinicians on the use of different antiseizure drugs in combination therapy is either very limited or lacking. In this study, the impact of lacosamide and perampanel alone and in combination was tested in corneal kindling model in mice, which is a cost‐effective mechanism for screening of antiseizure drugs.

**Methods:**

The impact of lacosamide (5 mg/kg) and perampanel (0.125 mg/kg) alone and their combination was tested in corneal kindling process (3‐mA current for 3 s applied twice daily for consecutive 12 days) in male BALB/c mice. Post‐kindling, mice were subjected to a battery of behavioral tests assessing anxiety, memory, and depression‐like behaviors. Brain tissues were then harvested for analysis of oxidative stress biomarkers.

**Results:**

Our results showed that the combination therapy of lacosamide and perampanel was more effective in reducing seizure progression than monotherapy of these drugs. Animals treated with combination therapy showed significant behavioral improvements, as reduced anxiety and depression were noticed, and their cognitive abilities were notably better compared to animals of all other groups. Moreover, biochemical assays of isolated brains from combination‐treated group revealed lesser amount of oxidative stress. In addition, outcomes of dual regime were comparable to the phenytoin in seizure control but showed superior benefits in mitigation of kindling‐prompted behavioral dysfunction and oxidative stress.

**Conclusions:**

This study suggests that the lacosamide and perampanel combination therapy worked noticeably better in halting the corneal kindling process in mice and improved the epilepsy‐associated psychiatric disorders that might be due to antioxidant effects of both drugs.

## Graphical abstract

Corneal kindling is a widely used model for assessment of antiseizures drugs. Lacosamide and perampanel in combination protected the mice from kindling‐induced seizures. This combination also reduced anxiety/depression‐like behavior in kindled mice. Treated animals had better memory retention and reduced oxidative stress.

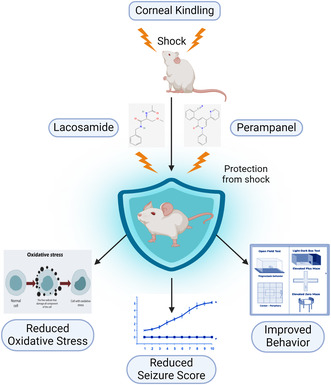

## 1 INTRODUCTION

Epilepsy is one of the most serious neurological disorders characterized by instinctive seizures, associated with cognitive and psychological consequences.^1^ In seizure, excessive and highly synchronous neuronal discharge occurs in the brain.^2^ The International League Against Epilepsy (ILAE) states that epilepsy is characterized by the presence of at least one of the following conditions: (1) minimum two unprovoked seizures that happen more than 24 h apart; (2) one unprovoked seizure and a likelihood of further seizures (≥60%) after two unprovoked seizures, following the next 10 years; (3) identification of epilepsy syndrome.^3^ Depending on the risks and etiological factors, the prevalence of epilepsy varies in different countries, but in men, its prevalence ratio is greater than women. The incidence of epilepsy in younger and elderly population is higher compared to other age groups.^4^ According to the report by the World Health Organization (WHO), about 50 million world's population is suffering from epilepsy, 80% of which are residing in low‐income countries.^5^ In epileptic patients, there are high incidences of injury and mortality when not treated optimally.^6^ Certain comorbidities such as anxiety, depression, dementia, and Alzheimer's disease are associated with epilepsy,^7^ and among these, at least one comorbidity is present in most of the epileptic patients.^8^ The prevalence of these comorbidities is eight times greater in the epileptic population compared to the general population.^7^

Seizure‐free life is the desired therapeutic outcome in the management of epilepsy. The main objective is to improve the patient's quality of life through seizure control and prevention of adverse drug events associated with the long‐term administration of antiepileptic medications.^9^ Antiseizure drugs, previously known as antiepileptic drugs, are regarded as the centerpiece for the treatment of epilepsy. Almost two‐thirds of epileptic patients are free from seizures by taking one or more antiseizure drugs.^10^ During the past two decades, many new drugs were approved for the treatment and management of epilepsy. Availability of these drugs and their long‐term safety profile increased the treatment options for epilepsy. Among these drugs, lacosamide (LAC), brivaracetam, rufinamide, and perampanel (PRP) are considered third‐generation antiepileptic drugs.^11^ LAC, an *N*‐acyl amino acid (available in both oral and intravenous forms), is approved in the USA and European Union as a monotherapy and adjunctive therapy for partial‐onset seizures in adults and children >4 years.^12^ It preferentially increases the delayed inactivation of voltage‐gated sodium channels and binds to the collapsin response mediator protein 2 (CRMP‐2), which is involved in the regulation of *N*‐type voltage‐gated calcium channels.^13^ But there is no clear evidence indicating direct binding of LAC to CRMP‐2.^14^ PRP is a new and first antiseizure drug that is involved in the glutamatergic transmission.^14^ It is a noncompetitive antagonist of α‐amino‐3‐hydroxy‐5‐methyl‐4‐isoxazolepropionic acid (AMPA) receptor and indicated as an adjunctive treatment of partial‐onset seizures with or without primary and secondary generalized seizures in adults and children ≥12 years.^15^

Kindling is a process in which the subconvulsive dose of any chemical or electrical stimulus leading to partial and generalized seizures is repeatedly administered.^16^ Previously in traditional kindling model, electric stimuli were given by unilateral depth electrodes that were implanted to hippocampus or amygdala.^17^ However, because electrode implantation is a very expensive and laborious technique, the use of this classic paradigm in screening novel chemical compounds for their anticonvulsant action is now on pause. This led to the creation of substitute techniques, including chemical and corneal kindling, to mimic the pathogenesis of epilepsy. Now a large number of researchers in the antiseizure drug screening programs use corneal kindling as an alternative to unilateral electrical kindling.^18^ Corneal kindling is a noninvasive procedure in which repeated electrical stimulations are given by saline‐soaked copper electrodes placed on cornea of the eye.^19^ According to Matang and Klitgaard's proposal, corneally kindled mice exhibit a very sensitive and sensible screening model that might improve the preclinical evaluation of the drug's efficacy and side effects when it comes to treating epilepsy.^20^

Although 70% of convulsive patients are managed using a single anticonvulsive drug, refractoriness encountered during treatment emphasizes the use of polypharmacy approach incorporating two or more drugs with a distinct mechanism of action.^21^ In view of this point, the current study aimed to evaluate the effect of LAC and PRP alone and in combination on kindling development and progression in corneally kindled mice. Subsequently, mice were tested for kindling‐associated neurobehavioral alterations through a battery of behavior experiments followed by a biochemical analysis of isolated brains for oxidative stress.

## 2 MATERIALS AND METHODS

### 2.1 Drugs and chemicals

PRP (Eisai Co, Japan), LAC (Venkata Narayana, India), and phenytoin (PHT) (Atco Laboratories Ltd., Pakistan) were used in this study. LAC at a dose of 5 mg/kg,^22^ PHT at a dose of 10 mg/kg,^16^ and PRP at a dose of 0.125 mg/kg^23^ were administered through the intraperitoneal route. LAC and PHT were dissolved in normal saline, whereas PRP was suspended in 1% tween 80. All these drugs were administered 40–45 min before the mice were given first electric shock. The dose for all the drugs used in this study was selected from the already‐published literature.

### 2.2 Animals

From the animal house of the Faculty of Pharmacy at Bahauddin Zakariya University in Multan, male mice of BALB/c strain, weighing between 25 and 35 g and aging between 10 and 12 weeks, were taken. The temperature and humidity levels in the housing were kept at 25 ± 2°C and 55%–65%, respectively, with a 12‐h light–dark cycle.

### 2.3 Corneal kindling procedure

Corneal kindling (Hugo Sachs Elektronik Harvard Apparatus, rodent shocker type 221, serial number 08080, Germany) in mice was performed according to the procedure given by Loscher and Potschka.^16^ In corneal kindling, mice were stimulated twice daily with the interstimulation interval of 6 h for 12 consecutive days. These electric stimulations were applied by placing saline‐soaked copper electrodes on cornea with 3‐mA current intensity for 3 s and pulse frequency of 50 Hz. Severity of epileptic seizures was scored according to the modified scoring system of Racine scale (1972): 1, modest facial clonus with blinking of eyes; 2, chewing, severe facial clonus, and head nodding; 3, forelimb clonus that may be unilateral or alternating; 4, rearing and forelimb clonus that is bilateral; 5, bilateral forelimb clonus with rearing and falling; 6, tonic hind limb extension. Mice exhibiting at least a stage 3 seizure after 12 days of the kindling treatment were classified as kindled mice, as reported earlier.^16^ Matange and Klitgaard reported that seizure severity reached a plateau after mice received electrical stimulation twice daily for 10–12 consecutive days. Increased stimulation did not make the seizures more severe.^20^

### 2.4 Animal grouping and treatment procedure

The sample size for each group was calculated by power analysis using the following equation: sample size = 2 SD^2^ (Z^α/2^ + Z^β^)^2^/d^2^, as described by Charan et al.,^24^ which was around 10 animals per group. For this study, a total of 60 healthy mice were randomly divided into six experimental groups, each containing 10 animals per group (as shown in Table 1). The mice were randomly allocated to these groups by independent blinded individuals who were unaware of the treatment protocols used in the study. First group received only a single daily dose of 0.9% normal saline intraperitoneally for 12 consecutive days; second group referred to as a diseased group received twice‐daily electrical stimulation for 12 consecutive days; third group named as PHT 10 (standard treatment group) received a single daily dose of PHT (10 mg/kg) for 12 days with twice‐daily electrical stimulation; fourth group named as LAC 5 group received a single daily dose of LAC (5 mg/kg) for 12 consecutive days with twice‐daily electrical stimulation; fifth group named as PRP 0.125 group received a single daily dose of PRP (0.125 mg/kg) for 12 consecutive days with twice‐daily electrical stimulation; and the sixth group named as LAC 5 + PRP 0.125 received the freshly prepared combination therapy of LAC (5 mg/kg) and PRP (0.125 mg/kg) for 12 consecutive days with twice‐daily electrical stimulation.

**TABLE 1:** Experimental groups.

Serial no.	Experimental groups
1	Healthy (only normal saline given for12 days)
2	Diseased (3 mA, 50 Hz, 3 s twice‐daily shock for 12 days)
3	PHT 10 (phenytoin 10 mg/kg once daily intraperitoneally with twice‐daily shock for 12 days)
4	LAC 5 (lacosamide 5 mg/kg once daily intraperitoneally with twice‐daily shock for 12 days)
5	PRP 0.125 (perampanel 0.125 mg/kg once daily intraperitoneally with twice‐daily shock for 12 days)
6	LAC 5 and PRP 0.125 combination once daily intraperitoneally with twice‐daily shock for 12 days

**FIGURE 1:**
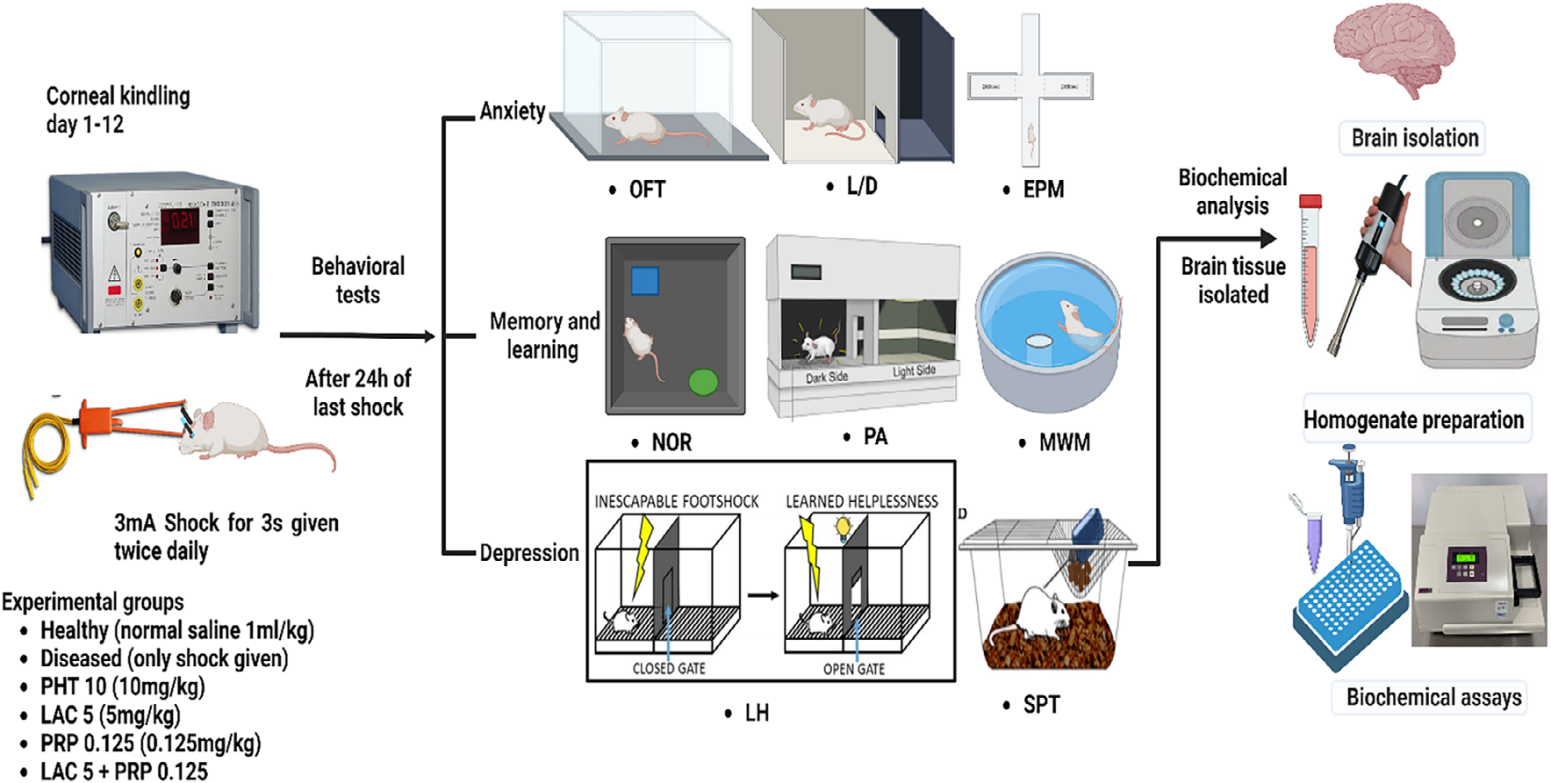
The schematic presentation of complete experimental layout. That experimental design involves corneal kindling, animal grouping, and neurobehavioral assessment for anxiety (open field test [OFT], light and dark test [L/D], elevated plus maze test [EPM]), depression (learned helplessness [LH] test, sucrose preference test [SPT]), and memory (passive avoidance [PA] test, novel object recognition [NOR] test, Morris water maze [MWM] test). After the behavioral testing, four animals per group were killed, their brains were removed, and biochemical analysis was performed to assess the levels of acetylcholine esterase (AChE), glutathione peroxidase (GPx), superoxide dismutase (SOD), malondialdehyde (MDA), and catalase. The figure was generated by biorender.com (agreement number: DA2703T2GM, dated June 30, 2024).

Drug treatment to mice was initiated on the first day of electrical stimulation and continued (for 12 days) until the corneal kindling protocol was completed. After 24 h of the last electrical stimulation, all the behavioral tests were performed (Figure 1). A Logitech web camera was placed above each test apparatus using a strong support system. This camera with its Logitech webcam software (installed in laptop) was used to record the animal's locomotor activity in each zone of test apparatus. The recorded videos were then analyzed using an ANY‐maze video tracking system within its 1‐month trial period. When all behavioral tests were completed, 4 animals from each group (total of 24 animals) were killed, their brains were removed and subjected to biochemical analysis.

### 2.5 Behavioral assessment for anxiolytic activity

#### 2.5.1 Open field test

After 24 h of the last electrical stimulation, an open field test (OFT) was conducted. This test was designed to assess the medication's impact on rodent anxiety, locomotion, and exploratory behavior. A mouse was placed in a void, square‐shaped open chamber with a dimension of 45 × 45 × 20 cm for 5 min. By tracking the mouse's number of entries at the center region and the overall amount of time spent there, the behavior was evaluated using ANY‐maze software.^25^ Animals that were more anxious stayed along the walls and spent a shorter time in an open arena. Each animal was tested once, and apparatus was cleaned with 70% isopropyl alcohol before placing the next animal to eliminate any type of smell clue from the previously tested animal.^26^

#### 2.5.2 Light and dark test

This test is designed to assess how a drug affects mice's anxiety‐like behavior. This test is based on rodents' innate resistance to entering a highly lit chamber and their impulsive exploratory behavior in unfamiliar environments.^27^ The apparatus used to conduct this test has two chambers: a light chamber measuring 21 × 21 × 25 cm and a dark chamber measuring 20 × 40 × 40 cm.^25^ There is a little aperture (7 × 6 cm) at the center of the two chambers for animal crossings, connecting the dark chamber (safe region) and the light chamber (aversive area). The mice were put in the light chamber and given 5 min to walk around freely between the two chambers. ANY‐maze software was used to record and evaluate the number of entries and the time spent in the light and dark zones.

#### 2.5.3 Elevated plus maze test

The elevated plus maze (EPM) test is utilized to assess mice's anxiety‐like behavior. The apparatus used for this test is a plus (+)‐shaped elevated maze that is 45 cm high from the ground and has two open arms that are perpendicular to the two enclosed arms (15 × 5.5 cm).^25^ Although most rodents naturally choose to live in enclosed settings, they also make an effort to explore open spaces.^28^ Each mouse was positioned in the middle of the EPM, facing the open arm. Each mouse was allowed to move freely in all the four arms of the test apparatus for 5 min. The number of entries and the time spent in the open and closed arms were recorded using a Logitech camera and later analyzed using ANY‐maze software.

### 2.6 Behavioral assessment for memory and learning

#### 2.6.1 Novel object recognition test

Various aspects of learning and memory, especially recognition memory in rodents, are evaluated using the novel object recognition (NOR) test. It is an inherent property of rodents to spend more time exploring a novel object compared to a familiar one. The purpose of this test is to assess the memory of rodents and determine whether they can differentiate between a familiar and a novel object.^28^ Each mouse was given two consecutive sessions that were performed in a squared‐shape box (40 × 40 cm) with walls (height 38 cm). In the training session (acquisition phase), two similar objects were placed in the square box, and mice were allowed to explore these objects for 5 min. In the testing session, mice were allowed to investigate the same box again for 5 min, except one of the objects that were previously explored was replaced by a different object. Mice preference for both objects (familiar and novel object) was noted, and then discrimination index was determined using the following formula.^29^ (Mice working memory shows improvement if discrimination index increased).

$$\begin{array}{c} \text{Discrimination index}=\\ \frac{\left(\text{time required for exploring}\;\mathrm{NO}\right)-\left(\text{time required for exploring}\;\mathrm{FO}\right)}{\left(\text{time required for exploring}\;\mathrm{NO}\right)+\left(\text{time required for exploring}\;\mathrm{FO}\right)}. \end{array}$$

where NO is the novel object and FO is the familiar object.

#### 2.6.2 Step‐through passive avoidance test

Gemini avoidance system (San Diego Instruments, USA) consists of two compartments (light and dark). Both compartments are interconnected by an electrically operated stainless steel door. Passive avoidance (PA) test is used to evaluate the rodent's memory and learning. This test comprises three sessions: one acquisition/training and two retention/test sessions. During the acquisition session, mice were placed in the brightly illuminated chamber and allowed to acclimate for 30 s. After this, the door was opened for 150 s to allow the mice to enter the dark chamber due to their inherent preference for dark space. When the mice entered the dark chamber, the door was closed, and a 0.3‐mA electrical foot shock was given for 2 s. Two retention sessions were performed immediately 1 and 24 h after the acquisition session to assess the mice's short‐ and long‐term memory. In the retention session, mice were placed inside the brightly illuminated chamber and allowed to acclimate this chamber for 10 s. After the acclimation period, the door was opened, and mice latency to enter the dark chamber was noted for 5 min. Mice with good memory show longer latency period to enter the aversive stimuli (dark chamber).^30^ The data of this test were taken from Gemini software (1.0.4 version).

#### 2.6.3 Morris water maze test

Long‐term spatial and learning memory in rodents is assessed by performing Morris water maze (MWM) test. The test apparatus used consists of a circular tub‐like tank with a 60 cm height and a diameter of 100 cm. Water opacified with a nontoxic opacifying dye was added into the tank up to 30 cm of height. The tank was divided into four quadrants (SE, NE, SW, and NW). A square‐shaped platform (12 × 12 cm) was placed 2 cm above the surface of water in the center of one quadrant (SW). To give the spatial learning point to the mice, the internal surface of the tank was mounted with various colorful geometrical shapes (proximal cues), and the same geometrical shapes were also mounted outside of the tank by wooden stands surrounding the tank (distal cues).^31^ This test was completed in 6 consecutive days: 2 days for training, 3 days for testing, and 1 day for probing. In the training days, the platform was kept visible in SW quadrant, and each animal was trained four trials a day to discover the platform within 2 min. If the animal failed to discover that visible platform within 2 min, then slight guidance was given with the help of a rod, and the animal was allowed to stay at the platform for about 10 s to develop the memory. In the testing days (next 3 days), the platform was submerged 1 cm below the water. Each animal was tested once a day for its ability to discover the hidden platform within 2 min, and escape latencies of these animals were noted. On the last day (probe day), the platform was removed from the tank, and each animal was allowed to swim for 2 min (1 trial/day) to note the number of entries and time spent by this animal in the targeted platform quadrant.^32^

### 2.7 Behavioral assessment for depression

#### 2.7.1 Learned helplessness test

Learned helplessness (LH) test is used to evaluate the depression in rodents. Gemini avoidance system (San Diego Instruments) was used for this test. It consists of two compartments (light and dark). Both compartments are interconnected by an electrically operated stainless steel door. In that test, mice were introduced in one compartment, with the gate between both compartments was closed. In the modified inescapable foot shock protocol, which is also called reduced type of LH protocol, 180‐ft shocks of 0.3 mA for 2 s were given to mice with 10‐s intershock interval. After 24 h of inescapable foot shocks, the mice were again placed in one compartment, and 30 escape trials were given. The total number of escapes from these 30 escape trials was recorded. Each trial duration was 26 s with 0.3‐mA foot shock and intertrial interval of 10 s. At the start of foot shock administration in escape trial, the gate between compartments opens to allow the mouse to escape foot shock. The trial in which mouse did not escape from the foot shock within 26‐s time limit was considered an escape failure. The mice with escape failures <15 were considered depression free. The mice with escape failures >15 were considered LH (mice with greater depression).^33^

#### 2.7.2 Sucrose preference test

Depression‐like behavior in animals is evaluated by the sucrose preference test (SPT), which is a reward based test.^34^ After 12 h of fasting, all animals were given free access to tap water (100 mL) and 1% sucrose solution (100 mL).^34^ After 24 h of free access to tap water and sucrose solution, the amount of tap water and sucrose solution consumed by animals was noted. Reduced consumption of sucrose solution indicates the disorder of reward behavior. SPT is one of the easy and nonaversive methods for assessing the rodent's depression‐like behavior. Percentage of sucrose preference is calculated by the following formula:

% sucrose preference = (sucrose solution intake)/(sucrose solution intake + water intake) × 100.

### 2.8 Biochemical analysis

#### 2.8.1 Preparation of brain homogenate for biochemical analysis

Four animals from each experimental group were chosen at random and beheaded (by cervical dislocation) to isolate their brains after the LH test. Using an IKA tech homogenizer, these separated brains were weighed and homogenized individually in 1:10 weight by volume of 0.1‐M phosphate buffer (pH = 7.4). The homogenized brains were centrifuged for 10 min at 4°C at 12000 rpm. After centrifugation, the supernatant was collected, separated into aliquots, and kept at −40°C until additional biochemical studies could be carried out. The levels of GPx, catalase, AChE, MDA, and SOD in the animal brain were measured using this collected supernatant. Protein contents, measured using Lowry's technique, were used to standardize the results.^25^

#### 2.8.2 Malondialdehyde assay

First a fresh mixture of 15% trichloroacetic acid (TCA) and 0.37% thiobarbituric acid (TBA) was prepared in equal proportion (1:1). Then the brain homogenate was added to that mixture to determine the MDA level. After being boiled for approximately 15 min in a water bath at 100°C, the reaction mixture was allowed to cool at room temperature. After cooling, the reaction mixture was centrifuged at 4°C for 10 min at 1000 g. After centrifugation, 200‐μL reaction mixture was loaded per well and the absorbance at 532 nm was noted using a spectrophotometer. The MDA concentration was given as micrograms of MDA/g of tissue.

#### 2.8.3 Superoxide dismutase assay

The reaction mixture was prepared by adding 50 μL of brain homogenate with 50 μL of 50 mM of sodium carbonate solution, 20 μL of 0.1 mM ethylenediaminetetraacetic acid (EDTA) (Sigma Aldrich, St. Louis, MO, USA), and 40 μL of 24 μM nitro blue tetrazolium (NBT). The reaction mixture was loaded into a 96‐well microplate. Further, to initiate the reaction (conversion of NBT into insoluble formazan), 40 μL (1 mM) hydroxylamine hydrochloride (HAC) solution was added into the reaction mixture. Absorbance of mixture was noted every 5 min up to 45 min at 570 nm using a spectrophotometer, and the activity was evaluated and given in units of SOD/mg of protein.

#### 2.8.4 Glutathione peroxidase assay

The experiment involved the mixing of 20 μL of brain homogenate with 20 μL of phosphate buffer, 10 μL of sodium azide (10 mM), 10 μL of hydrogen peroxide (2.5 mM), 20 μL of reduced glutathione (0.1 M) from Oakwood chemicals, and 20 μL of EDTA (0.8 mM). The reaction mixture was then incubated at 37°C for 15 min. To terminate the reaction, 40 μL of recently made 10% TCA was added to the reaction mixture. The mixture was then centrifuged for 5 min at 15000 rpm at 4°C. After centrifugation, 100 μL of the reaction mixture was mixed with 30 μL of disodium hydrogen phosphate (0.3 mM) and 70 μL of 5,5'‐dithio‐bis(2‐nitrobenzoic acid) (DTNB) (0.04%). Absorbance of this reaction mixture was noted at 412 nm in a microplate reader. GPx activity was measured using an extinction coefficient 14.15 mM^−1^ cm^−1^ and expressed in nmol/min/mg of protein.

#### 2.8.5 Catalase assay

An amount of 10 μL of brain tissue homogenate was taken in a microcentrifuge tube and mixed with 50 μL of phosphate buffer and 40 μL of 0.2 M hydrogen peroxide solution. This reaction mixture was incubated at 37°C for 90 s. Then 100 μL of 5% potassium dichromate acetic acid solution (freshly prepared by mixing 1 mL of 5% potassium dichromate solution and 3 mL of glacial acetic acid in the ratio of 1:3) was added in the reaction mixture to stop the reaction. The addition of potassium dichromate acetic acid solution caused the reaction mixture's color to change to blue. After that, the reaction mixture was heated for 15 min at 100°C during which time chromic acetate formed, and the reaction mixture's color changed from blue to green. Finally, the reaction mixture was centrifuged for 5 min at 4°C at 2500 g. After centrifugation, the reaction mixture's absorbance was measured in a microplate reader at 570 nm. The catalase activity was measured using the extinction coefficient 43.6 M^−1^ cm^−1^, and the results were reported in μmol/min/mg of protein.

#### 2.8.6 Acetylcholinesterase assay

For this assay, 40 μL of mice brain homogenate was taken in a microcentrifuge tube, and 20 μL of 0.01 M Ellman's reagent (DTNB) and 138 μL of phosphate saline buffer were added to it. All these reagents were mixed with the brain sample very well and kept at 25°C. After 2 μL of 0.075 M acetylthiocholine iodide was added, the absorbance at 412 nm was measured in a microplate reader every 2 min for 10 min. Using E = 13.6 mM^−1^ cm^−1^ helped with the calculation of the AchE activity in μmol/min/mg of protein.

### 2.9 Statistical analysis

Statistical analysis of data was performed by Graph pad prism of version 8. Prior to any statistical evaluation, data were normality assessed by the Shapiro–Wilk and Kolmogorov–Smirnov test. Behavioral and neurochemical data were evaluated by one‐way analysis of variance (ANOVA) post‐hoc Dunnett test, except for PA, MWM, and average seizure score. Average seizure score, step‐through and escape latencies in PA, and MWM, respectively, were analyzed using two‐way ANOVA followed by Dunnett's test. All data are expressed in ± standard error of the mean (SEM). If *p*‐value was <0.05, then the results were considered statistically significant.

## 3 RESULTS

### 3.1 Impact of LAC and PRP treatment on neurodynamic behavior of corneally kindled mice

**FIGURE 2:**
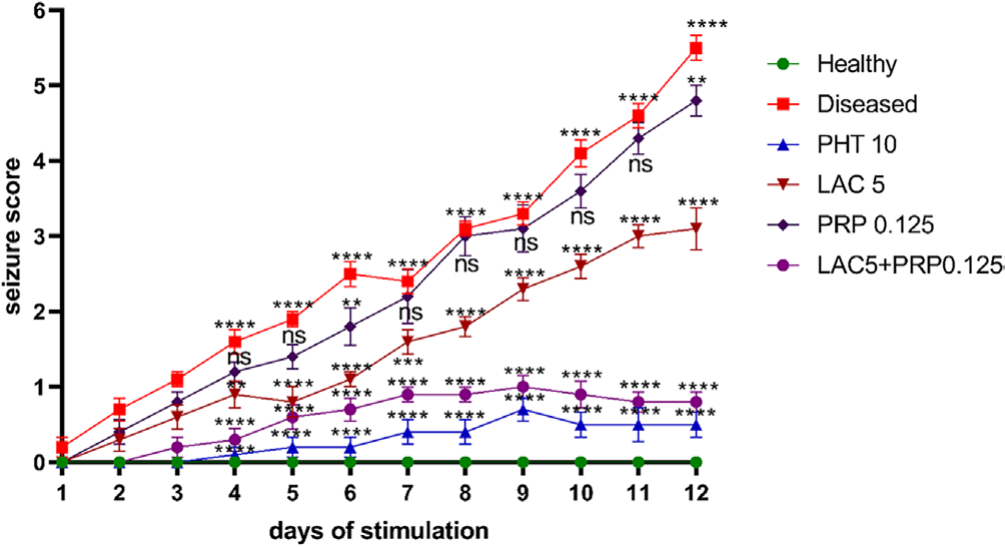
Impact of lacosamide (LAC) and perampanel (PRP) alone and in combination on seizure score of corneally kindled mice. LAC 5 mg/kg and PRP 0.125 mg/kg alone and in combination were given to mice daily for 12 days. The outcomes were compared with the group of diseased mice using a two‐way analysis of variance (ANOVA) evaluation, followed by Dunnett's test. The data for each group are shown as mean ± standard error of the mean (SEM) (*n* = 10), and the values *p < 0.05 and ***p < 0.001 are deemed statistically significant.

Continuous subconvulsive shock given to mice corneally resulted in increased convulsions' intensity and frequency. Seizure severities were assessed by modified Racine scale (1972). A shock of 3 mA for 3 s was given to mice corneally twice a day with 6‐h interstimulation interval for 12 days. This resulted in a gradual increase in seizure severity toward stage 5 and stage 6 in diseased group compared to the normal healthy group, as shown in Figure 2. A two‐way ANOVA was applied on obtained data that showed the gradual increase in seizure score from day 1 to day 12 of corneal stimulation (F [5, 648] = 614.4, *p* < 0.001) (Figure 2). Treatment with LAC and PRP alone suppressed the kindling progression compared to the diseased group, whereas LAC and PRP combination therapy exerted a more pronounced antiseizure effect compared to their single therapy and diseased groups. All the 10 mice in the combination therapy group remained seizure free, and their results were comparable with that of PHT.

### 3.2 Effect of LAC and PRP on anxiety‐like behavior in corneally kindled mice

The anxiolytic effects of LAC and PRP, individually and in combination, were evaluated in mice using OFT, light and dark (L/D) test, and EPM test.

**FIGURE 3:**
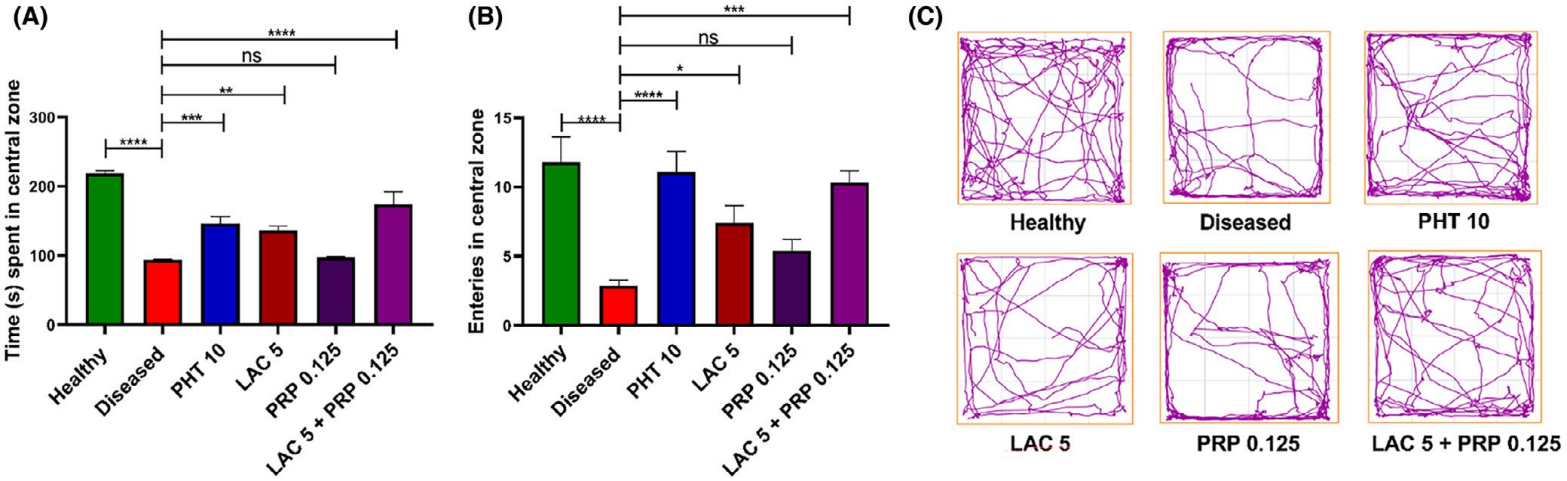
Effects of lacosamide (LAC) and perampanel (PRP) alone, and their dual therapy on anxiety of animals were noted by open field test (OFT). The test animal was placed in an open field arena for 5 min, and its anxiety was assessed by monitoring (A) time spent in center zone, (B) number of entries in center zone, and (C) locomotor activity of the animal by track plots. After a one‐way analysis of variance (ANOVA) and Dunnett's test, the findings were compared with the diseased group animals. Each group's mean ± standard error of the mean (SEM) (*n* = 10) is used to represent the results, with *p < 0.05, **p = 0.01, and ***p < 0.001 being statistically significant and ns as statistically nonsignificant.

In OFT, each mouse was given the opportunity to explore the open maze to assess behaviors such as anxiety and locomotor activity. The statistically significant differences among all groups for the time spent in the center zone (F [5, 54] = 28.16, *p* < 0.001) and the number of entries in the center zone (F [5, 54] = 8.717, *p* < 0.001) are illustrated in Figure 3A,B, respectively. The diseased group mice showed more anxiety compared to healthy and treatment groups. Mice treated with LAC monotherapy showed enhanced preference for central zone (*p* = 0.042), whereas the outcomes of PRP monotherapy were nonsignificant (*p* = 0.444). The results of LAC and PRP combination therapy were statistically better (*p* = 0.003), as the mice treated with this combination therapy showed greater preference toward the central zone.

**FIGURE 4:**
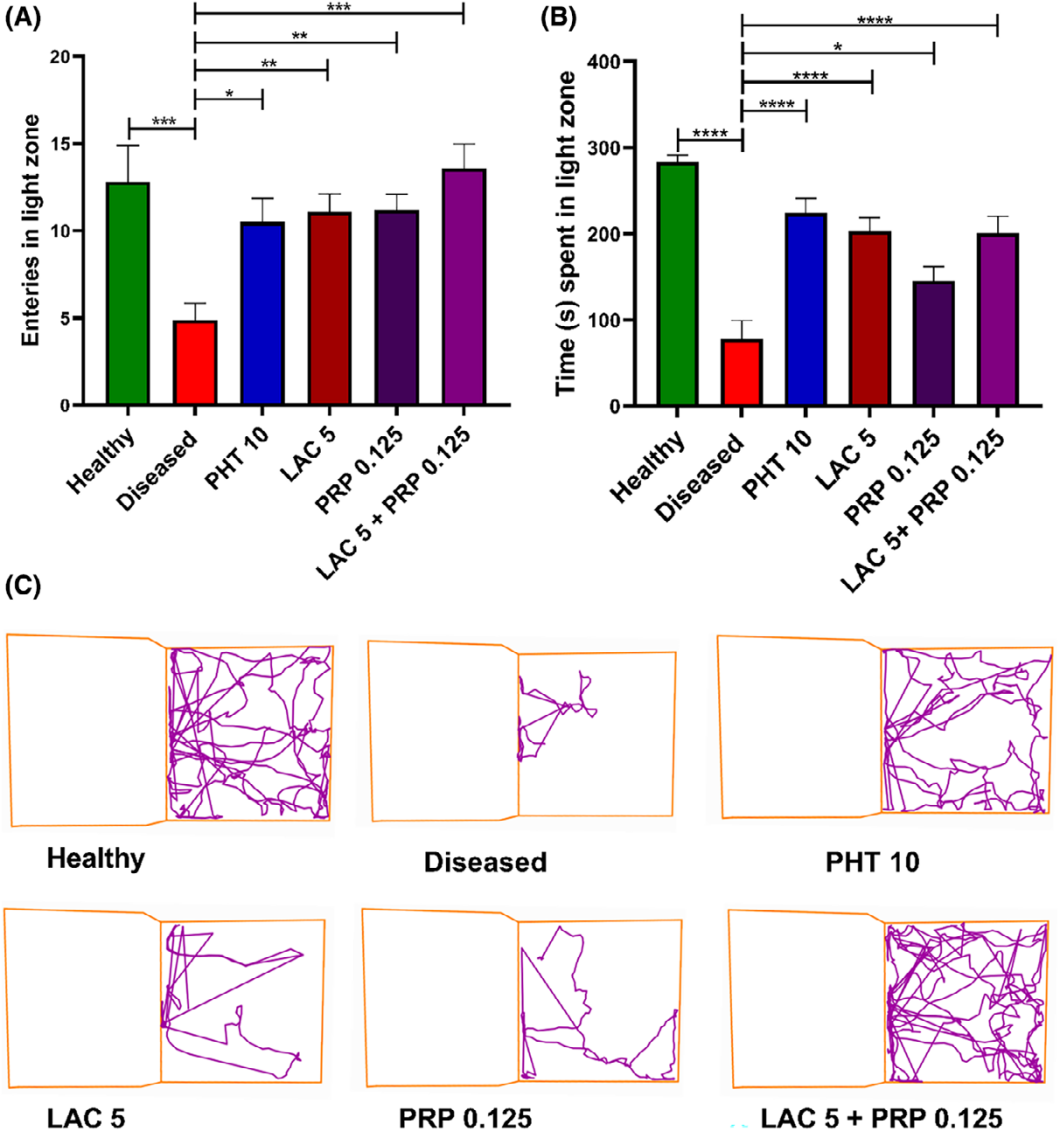
Post‐kindling, the anxious behavior of animals treated with lacosamide (LAC) and perampanel (PRP) alone, as well as their combination, was evaluated using the light and dark (L/D) test. In that experiment, animals were allowed to move freely in light and dark boxes for 5 min. Their (A) number of entries in the light zone, (B) time spent in the light zone, and (C) track plots in both the light and dark zones were monitored. The outcomes were compared with the group of diseased mice using a one‐way analysis of variance (ANOVA) and Dunnett's test. Each group's mean ± standard error of the mean (SEM) (*n* = 10) is used to represent the results, with *p < 0.05, **p < 0.01, and ***p < 0.001 being statistically significant and ns as statistically nonsignificant.

In L/D test, mice were free to explore the bright and dark areas. The number of times these mice entered the bright and dark zones and the amount of time they spent there, respectively, were utilized for assessing their level of anxiety. Analysis of these parameters showed significant variation in the number of entries among the groups in the light zone (F [5, 54] = 5.128, *p* = 0.001), as shown in Figure 4A, and the amount of time spent in the light zone (F [5, 54] =17.42, *p* < 0.001), as indicated in Figure 4B. Compared to the healthy group, the diseased group mice had a reduced duration of stay in the light zone (*p* < 0.001) and were more reluctant to enter the illuminated area. Mice treated with LAC and PRP monotherapy spent greater time in the bright zone (*p* < 0.001 and *p* = 0.028, respectively). Those mice treated with LAC and PRP combination therapy were less anxious or affected by the corneal kindling, as they spent longer time in the light zone (*p* < 0.001). The outcomes of combination therapy were comparable to that of PHT.

**FIGURE 5:**
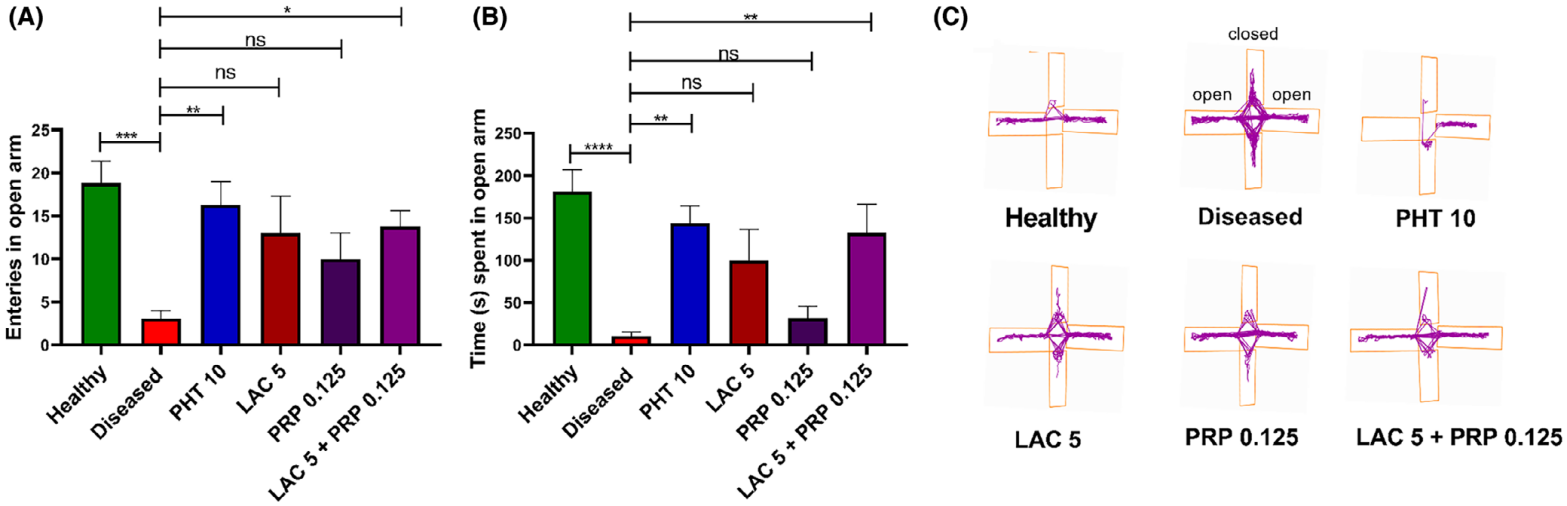
Evaluation of the effects of lacosamide (LAC) and perampanel (PRP) monotherapy and their dual therapy on anxiety of animals using elevated plus maze (EPM) test. Animals were allowed to move freely in plus‐shaped apparatus for 5 min, and their anxious behavior was assessed by monitoring (A) the total entries in open arm, (B) duration of the time spent in open arm, and (C) track plots in both arms. The outcomes were compared with the diseased group animals using a one‐way analysis of variance (ANOVA) and Dunnett's test. All results are reported as mean ± standard error of the mean (SEM) (*n* = 10) for each group; ns is regarded as statistically nonsignificant, and *p < 0.05, **p < 0.01, and ***p < 0.001 are statistically significant.

In the EPM test, a significant difference was observed among all groups in the number of entries in open arms (F [5, 54] =3.952, *p* = 0.004) and the time spent in open arms (F [5, 54] = 7.050, *p* < 0.001), as shown in Figure 5A,B, respectively. The diseased group mice showed more anxiety as they spent little time in open arms (*p* < 0.001) compared to healthy and other treatment groups. Outcomes of mice treated with LAC (*p* = 0.061) and PRP (*p* = 0.964) alone were nonsignificant as they also spent less time in open arms. But the outcomes of LAC and PRP combination therapy were more significant (*p* = 0.005), as that group stayed in open arm for much greater duration compared to all other groups, except healthy and PHT 10 groups. The number of entries in open arms was also greater in mice treated with combination therapy (*p* = 0.035) compared to diseased group mice and mice treated with single therapy.

### 3.3 Impact of LAC and PRP treatment on learning and memory in corneally kindled mice

**FIGURE 6:**
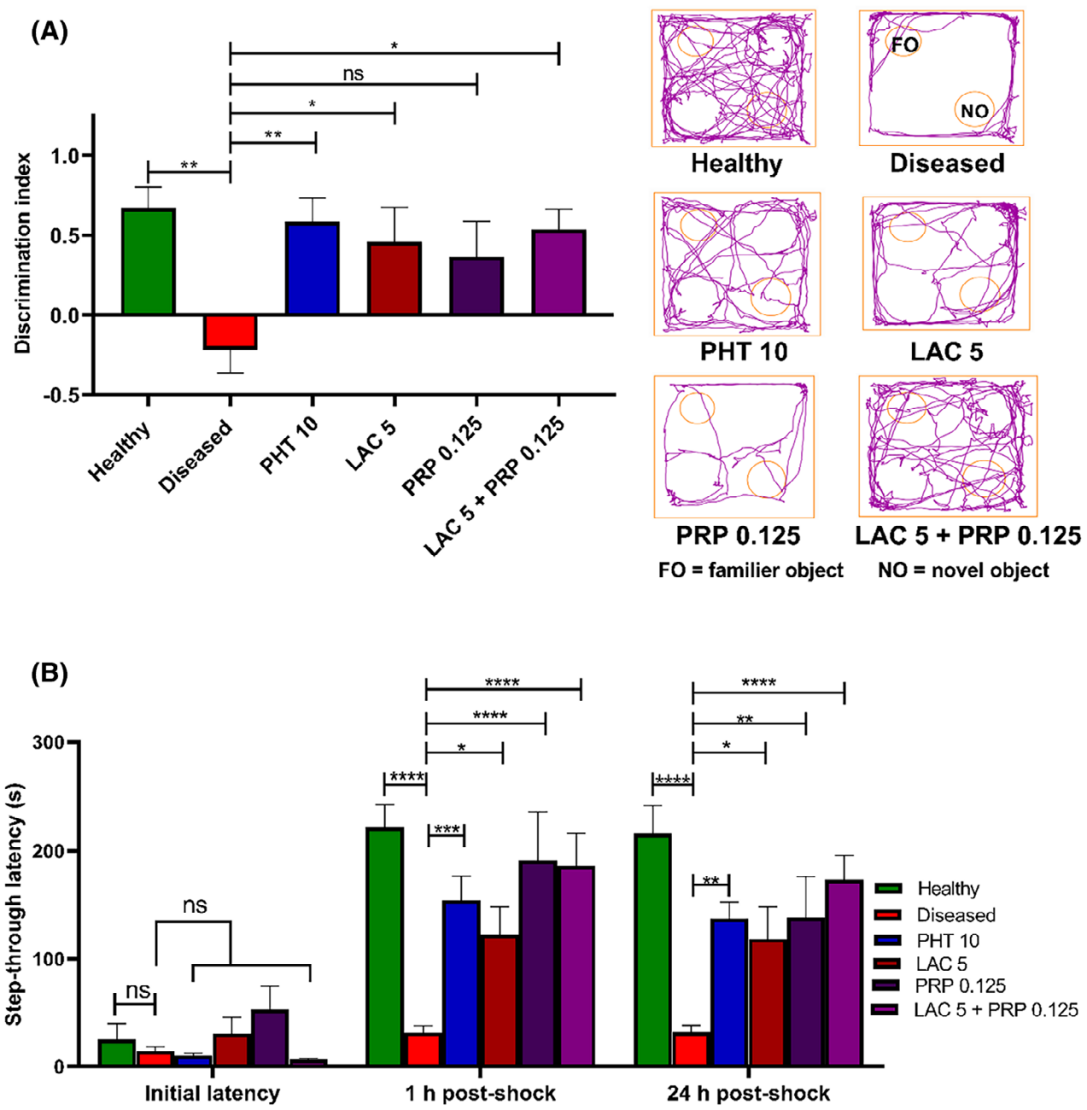
Evaluation of the impact of lacosamide (LAC) and perampanel (PRP) alone and their dual therapy on memory and learning behavior of corneally kindled mice through novel object recognition (NOR) and passive avoidance (PA) tests. In NOR apparatus, ability of the mice to differentiate between novel object and previously familiarized object was assessed for 5 min using (A) discrimination index and tracking plots. The results of NOR test were analyzed by one‐way analysis of variance (ANOVA) followed by Dunnett's test and compared with the diseased group animals, whereas in PA test, mice were trained in PA apparatus for remembrance of aversive stimuli. Mice cognitive memory was evaluated using avoidance of aversive stimuli in two 5‐min sessions performed after 1 and 24 h of initial training. Mice cognitive ability was expressed by (B) step‐through latency. The outcomes of that test were compared to diseased group animals after a two‐way ANOVA and Dunnett's test were performed. All results are expressed as mean ± standard error of the mean (SEM) (*n* = 10) for each group; ns is regarded as statistically nonsignificant and *p < 0.05, **p < 0.01, and ***p < 0.001 as statistically significant.

The neurocognitive effects of LAC and PRP were explored in mice utilizing NOR, passive avoidance (PA) test, and MWM test. In the NOR test, mice's memory was assessed by evaluating their capacity to distinguish between a novel and previously recognized item. Mice with good memories typically sought to investigate the novel object after recalling the previous one. Figure 6A illustrates a significant difference between the groups observed through one‐way ANOVA (F [5, 54] = 3.543, *p* = 0.008). Compared to the healthy group and other treatment groups, the diseased group mice showed a poor discrimination index (*p* = 0.002). The results of LAC single therapy and combination therapy (LAC + PRP) were statistically significant as they demonstrated greater discrimination indexes (*p* = 0.029 and *p* = 0.012, respectively), whereas the results of PRP‐treated mice were not statistically significant (*p* = 0.074).

In the PA test, two‐way ANOVA findings exhibited significant differences between the groups for step‐through latencies (F [5, 162] =11.38, *p* < 0.001), as shown in Figure 6B. Animals were first placed in the lighted compartment during the training session. After 30 s of acclimatization, the animals were moved into the shock zone, or dark compartment, and no discernible differences were observed between the groups. After 1 h of shock, animals were tested again to check the remembrance of aversive stimuli. Compared to healthy animals, diseased animals exhibited shorter latencies (*p* < 0.001) in exiting the light compartment and entering the dark compartment, indicating worse memory of unpleasant stimuli. Compared to diseased animals, the animals treated with a combination treatment (consisting of LAC and PRP), PRP alone exhibited greater latencies toward the dark compartment (*p* < 0.001) because of their ability to recall the aversive stimuli. Animals treated with LAC alone also showed increased latencies (*p* = 0.022) toward the dark compartment compared to diseased animals. But LAC‐treated group's latency was shorter than other treatment groups. After 24 h of the last shock, mice were again tested to evaluate their long‐term memory and recalling capacity. In this second test session, diseased mice again revealed poor latencies (*p* < 0.001) toward the dark compartment, as they rapidly entered in that compartment compared to healthy mice. Mice treated with LAC and PRP combination (*p* < 0.001) or monotherapy of these drugs, that is, LAC (*p* = 0.032) or PRP (*p* = 0.005) alone had extended latency, toward the dark compartment. The results of PHT‐treated mice (*p* = 0.006) were similar to those treated with PRP alone.

**FIGURE 7:**
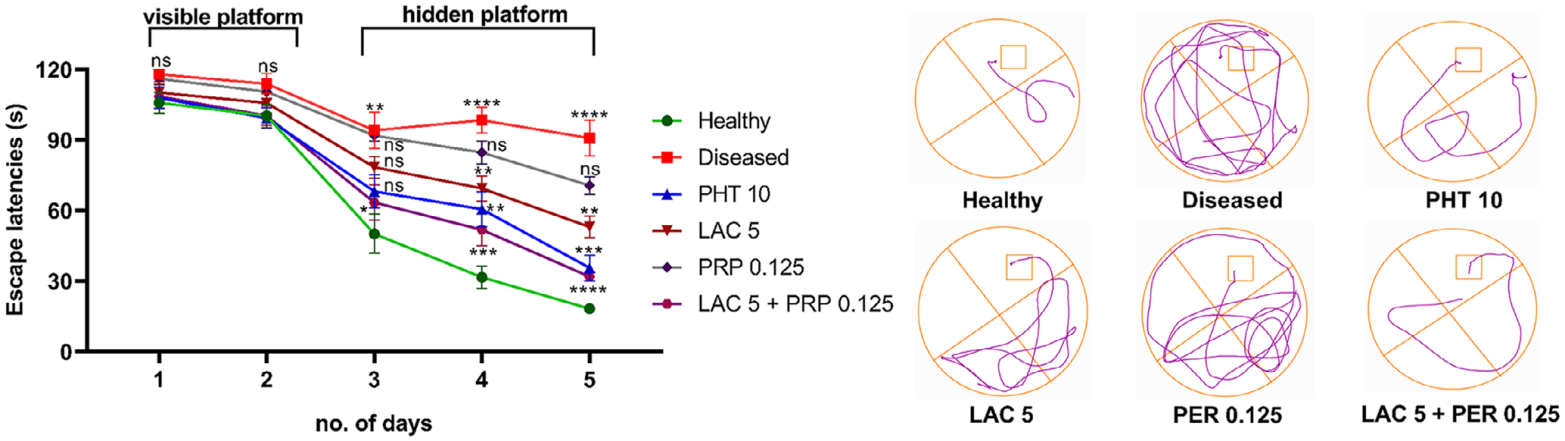
Assessment of impact of lacosamide (LAC) and perampanel (PRP) alone and in combination on memory of corneally kindled mice using Morris water maze (MWM) test. The animal was allowed to locate the platform for 2 min in MWM for 5 consecutive days. The memory and learning behavior of mice were evaluated through the escape latencies to enter the platform zone. The results were analyzed using two‐way analysis of variance (ANOVA) followed by Dunnett's test. All data are presented in mean ± standard error of the mean (SEM) (*n* = 10) for all groups. *p < 0.05, **p < 0.01, ***p < 0.001 are considered statistically significant, and ns is considered statistically nonsignificant.

The MWM test was used to analyze the working and reference memory of mice for 6 consecutive days. Two‐way ANOVA exhibited a marked intergroup difference in escape latencies for the first 5 days (F [5, 54] =13.71, *p* < 0.001), as shown in Figure 7. When we compared the escape latencies of the fifth day, diseased group showed poor remembrance of platform location as their escape latencies (*p* < 0.001) were longer than those of the healthy group. Mice treated with monotherapy of LAC showed decreased escape latencies (*p* = 0.003) and less swimming time in locating the hidden platform compared to diseased group mice, whereas the results of mice treated with PRP alone were nonsignificant (*p* = 0.119). The combination therapy of LAC and PRP showed enhanced cognition memory in locating the submerged platform as their escape latencies (*p* < 0.001) were shorter than those of the diseased group mice, thus validating the better outcomes of combination therapy over monotherapy.

**FIGURE 8:**
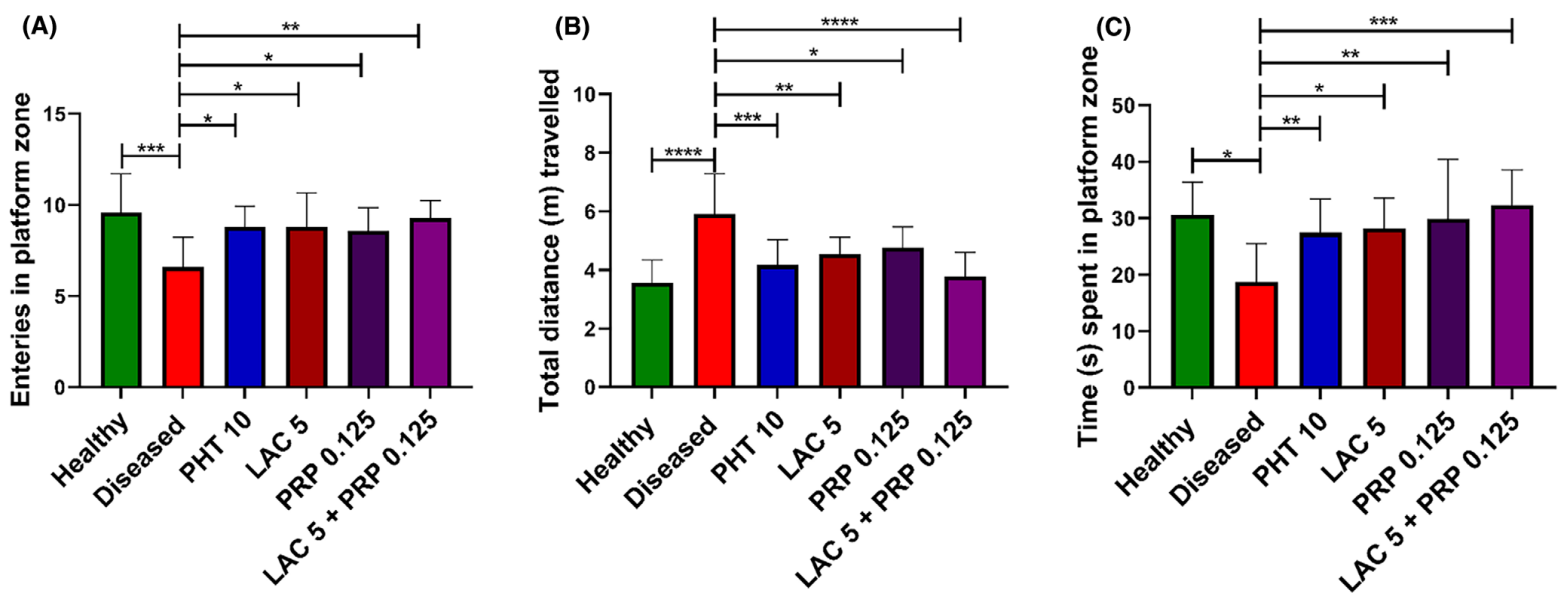
Effects of lacosamide (LAC) and perampanel (PRP) monotherapy and their dual therapy on cognitive capacity of corneally kindled mice were evaluated during the probe day using Morris water maze (MWM) test. Mice memory and learning were assessed by monitoring (A) the number of entries in the platform zone, (B) total distance traveled, and (C) the time spent in the platform zone. The results were analyzed by one‐way analysis of variance (ANOVA) followed by Dunnett's test and compared with the diseased group animals. All data are presented in mean ± standard error of the mean (SEM) (*n* = 10) for all groups, whereas *p < 0.05, **p < 0.01, ***p < 0.001 are considered statistically significant.

At the sixth day of the MWM test, which is the probe day, all mice were assessed for their cognitive capacity by observing their remembrance of the rescue zone in which the platform was submerged (SW zone). One‐way ANOVA showed a marked intergroup difference for the number of entries (F [5, 54] = 4.608, *p* = 0.001), total distance traveled (F [5, 54] =8.943, *p* < 0.001), and the time spent (F [5, 54] = 4.708, *p* = 0.001) in the target zone, as shown in Figure 8A–C, respectively. The diseased group animals showed poor remembrance of rescue zone, as their number of visits in target zone (*p* = 0.003) was less than the healthy animals. Animals treated with LAC and PRP monotherapies showed a greater number of entries (*p* = 0.011 and *p* = 0.024, respectively) in the target quadrant compared to diseased animals. Animals treated with LAC and PRP combination therapy showed the highest number of entries (*p* = 0.001) in the target quadrant among all the treated groups, exhibiting better retention of reference memory compared to diseased animals and other treatment groups.

These results were further confirmed by analyzing the total distance traveled by the mice and the time they spent in the rescue zone in search of the platform. The diseased group showed thigmotaxic and impaired memory behavior as they continued to move throughout the water tank. On the probe day, the total distance traveled by diseased animals was greater (*p* < 0.001) than that by the healthy animals. Animals treated with LAC and PRP monotherapies or combination therapy were protected from thigmotaxic behavior (*p* = 0.005 and *p* = 0.025, respectively), as the total distance traveled by these animals was lesser (*p* < 0.001) than that by the diseased animals.

Similarly, diseased animals spent less time (*p* = 0.002) in target quadrant compared to healthy animals. Compared to the diseased group animals, mice treated with LAC, PRP, or their combination exhibited substantially longer swim durations in the target zone (*p* = 0.017, *p* = 0.003, and *p* = 0.001), displaying better cognition and memory.

### 3.4 Impact of LAC and PRP therapy on depression‐like behavior in corneally kindled mice

**FIGURE 9:**
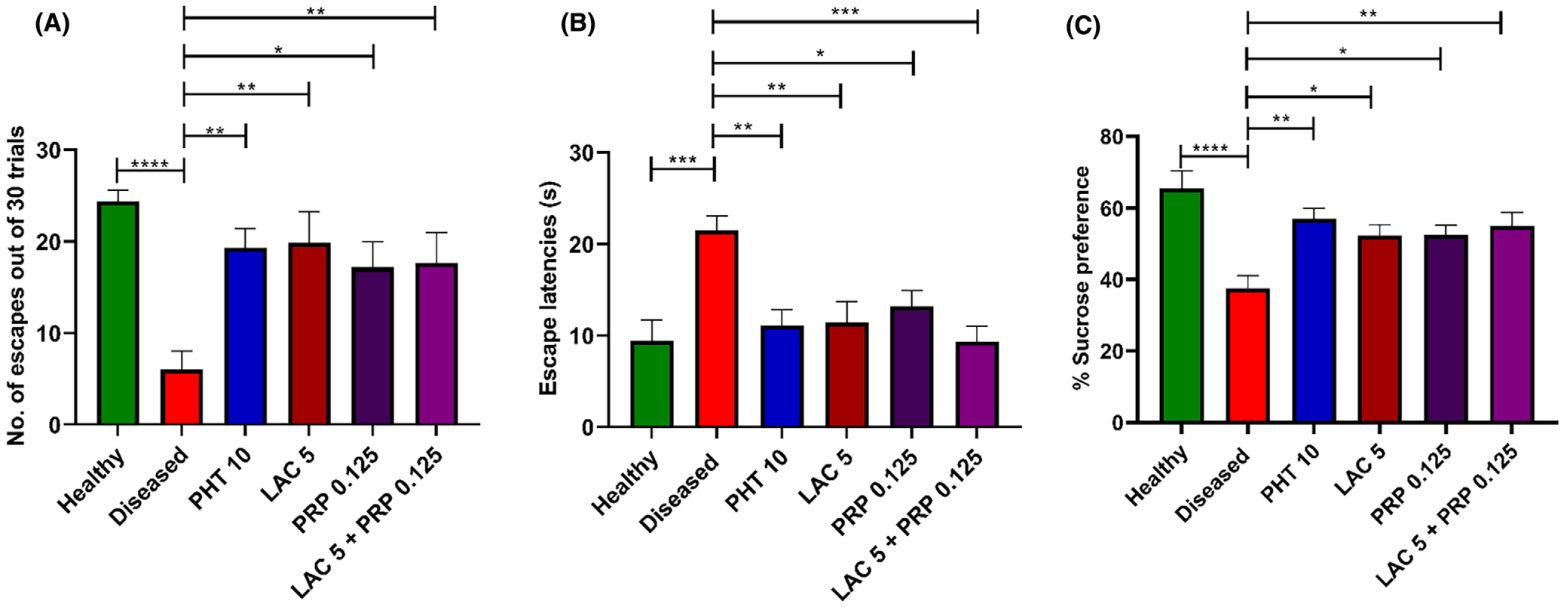
Depressive behavior of corneally kindled mice treated with lacosamide (LAC), perampanel (PRP), and their combination therapy was evaluated using learned helplessness (LH) and sucrose preference (SP) tests. In LH test, mice's depressive behavior was determined by monitoring (A) the number of escapes out of 30 trials and (B) escape latencies. In SP test, animals' depression was measured by their (C) percentage of SP within 24 h for 1% solution of sucrose. The outcomes of these tests were examined using a one‐way analysis of variance (ANOVA) followed by Dunnett's test, with a comparison made to diseased animals. For each group, the results are reported as mean ± standard error of the mean (SEM) (*n* = 10), with *p < 0.05, **p < 0.01, and ***p < 0.001 being statistically significant and ns as statistically nonsignificant.

The efficacy of LAC and PRP alone and their combination therapy in mitigating depression‐like behavior was evaluated in mice using LH test and SPT. Results of LH test showed a marked intergroup difference in the number of escapes out of 30 trials when analyzed statistically (F [5, 54] = 5.695, *p* = 0.003). Corneally kindled mice showed a smaller number of escapes (*p* < 0.001) compared to healthy mice, because the diseased mice learned helplessness (undergoes to depression), as shown in Figure 9A. The outcomes of mice treated with LAC alone and PRP alone were statistically significant as they showed a large number of escapes (*p* = 0.002 and *p* = 0.014 respectively) compared to the diseased group mice. Mice treated with combination therapy of LAC and PRP and PHT showed a larger number of escapes, and their results were also statistically significant (*p* = 0.009 and *p* < 0.003, respectively). A marked intergroup difference in escape latencies (F [5, 54] = 6.638, *p* < 0.001) was also observed, as shown in Figure 9B. The diseased group mice revealed greater escape latencies (*p* = 0.002) compared to the healthy mice, whereas the escape latencies of mice treated with combination therapy of LAC and PRP or monotherapy of LAC and PRP were significantly lower (*p* = 0.002, *p* = 0.002, and *p* = 0.013, respectively) than the diseased mice. Mice treated with PHT also exhibited shorter escape latencies (*p* = 0.001).

In SPT, statistical analysis showed a marked difference in the consumption of sucrose in the diseased group compared to all other treatment groups (F [5, 54] = 6.415, *p* < 0.001), as shown in Figure 9C. Corneal kindled mice presented marked anhedonia, a classical characteristic of depressive‐like behavior compared to the healthy group, as these animals showed greater proclivity toward sweetened sucrose solution (*p* < 0.001). The preference toward sucrose solution was highest in animals treated with PHT (*p* = 0.002) followed by LAC‐ and PRP‐based combination therapy (*p* = 0.006), PRP (*p* = 0.021), and LAC (*p* = 0.023) monotherapies.

### 3.5 Impact of LAC and PRP treatment on kindling‐induced oxidative stress

**FIGURE 10:**
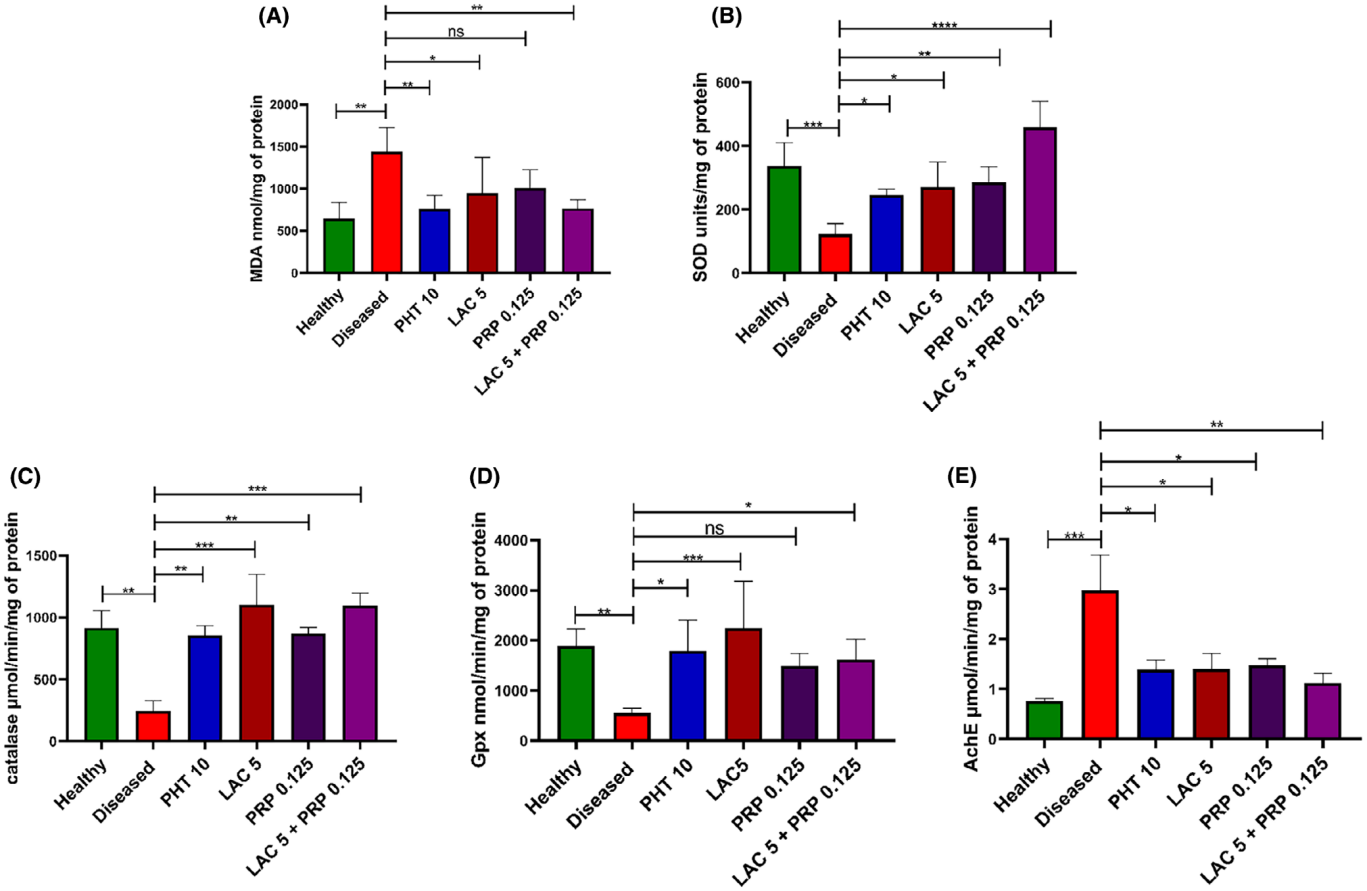
Biochemical analysis of isolated brain tissues from mice demonstrated the effects of lacosamide (LAC), perampanel (PRP), and their combination therapy on different biomarkers of oxidative stress. In that biochemical analysis, levels of (A) malondialdehyde, (B) superoxide dismutase, (C) catalase, (D) glutathione peroxidase, and (E) acetylcholinesterase were determined. The outcomes were compared with diseased animals after a one‐way analysis of variance (ANOVA) and Dunnett's test were performed. All results are reported as mean ± standard error of the mean (SEM) (*n* = 10) for each group; ns is regarded as statistically nonsignificant, and *p < 0.05, **p < 0.01, and ***p < 0.001 are regarded as statistically significant.

MDA is considered one of the well‐known biomarkers to evaluate the oxidative stress associated with epilepsy.^35^ Statistical analysis revealed a marked intergroup difference in MDA levels in brain homogenates (F [5, 18] = 5.079, *p* = 0.005), as shown in Figure 10A. The diseased group mice showed increased oxidative stress indicated by a higher level of MDA (*p* = 0.001) compared to the healthy mice. Increased oxidative stress led to enhanced lipid peroxidation and neuronal damage in the diseased mice. Levels of MDA were significantly reduced in mice treated with combination therapy of LAC and PRP and LAC alone (*p* = 0.006 and *p* = 0.049, respectively). Results of PRP‐treated mice were nonsignificant (*p* = 0.097).

SOD is well known for its antioxidative and neuroprotective activity.^36^ Statistical analysis of SOD activity was significantly different among all the groups with (F [5, 18] = 13.16, *p* < 0.001), as shown in Figure 10B. The diseased mice showed marked depletion of SOD activity (*p* = 0.004) compared to the healthy mice. Mice treated with LAC monotherapy and PRP monotherapy revealed less oxidative stress, as the level of SOD was markedly enhanced (*p* = 0.012 and *p* = 0.006, respectively) in these animals. The level of SOD activity significantly increased (*p* < 0.001) in mice treated with the combination therapy of LAC and PRP. These results showed the importance of combination therapy over monotherapy, demonstrating better outcomes.

Similarly, the catalase enzyme is present in most of the living organisms, which provides protection from the oxidative damage caused by reactive oxygen species. Statistical analysis showed a marked intergroup difference in the level of catalase activity with (F [5, 18] = 7.571, *p* = 0.001), as shown in Figure 10C. Significantly decreased catalase activity (*p* = 0.003) was observed in diseased group compared to the healthy group. Mice treated with monotherapy of LAC and PRP showed less oxidative stress as the catalase levels were high (*p* = 0.002 and *p* = 0.005, respectively) in them compared to the diseased mice. The level of catalase activity was upregulated in mice treated with the combination of LAC and PRP (*p* = 0.003) compared to the diseased mice.

The GPx enzyme has peroxidase activity that protects cells from oxidative stress. Results of ANOVA showed a significant intergroup difference in the GPx level with (F [5, 18] = 4.902, *p* = 0.005), as shown in Figure 10D. In the diseased group mice, large neuronal damage was observed due to oxidative stress caused by a low level of GPx (*p* = 0.008) compared to the healthy mice. The mice treated with monotherapy of LAC and dual therapy of LAC and PRP showed enhanced neuroprotection and antioxidant potential compared to the diseased mice because of the improved level of GPx activity (*p* = 0.001 and *p* = 0.038, respectively). The results of mice treated with PRP alone were nonsignificant (*p* = 0.077).

AchE enzyme is involved in the degradation of acetylcholine. Acetylcholine is a crucial neurotransmitter of central nervous system, playing an important role in learning and memory.^37^ Higher levels of AchE are often correlated with decreased acetylcholine levels and poor prognosis of epilepsy and learning. Our biochemical analysis showed a marked intergroup difference in AchE activity (F [5, 18] = 5.032, *p* = 0.005), as indicated in Figure 10E. In the diseased mice, AchE activity was higher (*p* = 0.001) than the healthy mice, as AchE reduces the brain's cholinergic anti‐inflammatory response and increases predisposition to epilepsy.^38^ However, the AchE activity was downregulated in mice treated with LAC alone (*p* = 0.017), PRP alone (*p* = 0.023), and their combination (*p* = 0.005) compared to the diseased mice.

## 4 DISCUSSION

Although most epilepsy patients are treated with a single standard antiseizure drug, some either do not respond to the prescribed drug or show resistance toward that drug gradually.^39^ Despite increased understanding of the mechanism of seizures, epileptogenesis, and the mode of action of antiseizure drugs, still standard treatment of epilepsy fails in about 30% of patients, and they become refractory to antiseizure drugs.^40^, ^41^, ^42^ In such situations, most physicians prescribe alternative monotherapy to control the convulsive attacks; however, complete suppression of seizures is rarely achieved, even if the antiseizure drug is prescribed at a maximum dose.^42^ So, in such a case, a second antiseizure drug is often added to the therapeutic regimen to get a sustained antiseizure response.^43^ Anticonvulsants are known to impede epileptogenesis when used in polypharmacy, because their multiple targets increase the total effectiveness of each medication. Unfortunately, the danger of polypharmacy in epilepsy that is drug‐resistant is unavoidable due to the increased risk of drug‐associated neuropsychiatric adverse effects from taking many medications at once. To address this problem, combination therapy's potential to suppress seizures must be investigated at low doses to lower the possibility of side effects.

Polytherapy is advantageous as it helps in reducing the dose, minimizes undesirable effects of drug, and provides better protection against different types of seizures.^43^, ^44^ In our study, we compared the antiepileptic effects of LAC, PRP, and their combination on the neurodynamic behavior of corneally kindled mice. To the best of our knowledge, this study is the first of its kind to investigate the combined effect of PRP and LAC in corneally kindled model of epilepsy. The two anticonvulsant medications have distinct modes of action, and the study postulated that taking them together might lessen the risk of seizures and improve anticonvulsant effect.

The most important feature of kindling is the sprouting of mossy fibers in the dentate gyrus, which is frequently correlated with mammalian epileptic brain.^45^ Hippocampus plays a very important role in pathogenesis of seizures and epilepsy.^46^ Recent studies show that subconvulsive electrical stimulation results in sprouting of axons in the hippocampus of rodents.^47^ In addition, corneally kindled seizures induced astrogliosis in dentate gyrus and CA1 region of hippocampus without neuronal loss.^48^ Corneal kindling is used as a chronic epilepsy model, as it increases susceptibility to seizures and induces cellular and molecular alteration that leads to oxidative stress and neurodegeneration.

The first step of the study involved the evaluation of the anticonvulsant effect of test drugs, both used alone and in combination, on corneally kindled mice. Subconvulsive electrical stimulation in mice via cornea increases severity of seizures compared to the healthy mice. However, mice treated with LAC, PRP, and their combination exhibited comparatively reduced severity of seizures because these drugs hindered the process of kindling. Mice treated with LAC and PRP alone exhibited stage 3–4 seizures, whereas seizure progression was significantly halted in mice treated with the combination of LAC and PRP, as they exhibited maximally up to stage 2 seizures of modified Racine score. The diseased mice (those that only received electrical shock) started to have stage 1–2 seizures on the 5th and 6th days of corneal kindling, and the severity of these seizures reached stage 6 until the 12th day (the last day) of corneal stimulation/kindling. However, individual administration of LAC and PRP in mice increased the threshold of seizure and produced significant latency in the seizure's development, whereas co‐administration of PRP and LAC was more efficacious in reducing the seizure progression compared to their monotherapy. PHT was used as a standard therapy, as it is a voltage‐gated sodium channel blocker; it keeps the sodium channel in its inactive state while extending the refractory period of neurons.

However, PHT nowadays is used less commonly due to its narrow therapeutic window, strong protein binding, nonlinear pharmacokinetics, and interactions with other drugs and diseases. PHT has a significant risk of side effects, including peripheral neuropathy, cerebellar impairment, allergic responses, pseudolymphomas, and gingival hyperplasia. Over time, using PHT may exacerbate abrupt seizures; however, seizures may also worsen after the PHT is discontinued.^49^, ^50^

The AMPA antagonist PRP is authorized for use, both alone and in combination, to treat partial and generalized seizures. PRP efficacy in seizure models of rodent was first documented by Hanada et al. and subsequently verified by Lee et al.^51^, ^52^ Hanada et al. conducted research in which they evaluated PRP's wide range of antiseizure activity using several models, such as the pentylenetetrazole (PTZ) induced kindling model. In this model PRP was administered at a dose range of 0.75–3 mg/kg.^53^ Another research by Russmann et al. found that using doses of 1.5, 2.25, and 3 mg/kg in rats had a dose‐dependent impact on amygdala kindling.^54^

For adults and children >4 years with partial‐onset seizures, LAC is licensed in the EU and the USA as both a monotherapy and an adjunctive treatment.^12^ Our findings concerning LAC are corroborated by a prior study conducted by Stohr et al., in which mice and rats that were given maximal electroshock seizure, hippocampal, and PTZ kindling experienced less seizure when treated with LAC monotherapy at a dose range of 3.9–4.5 mg/kg.^22^ Another clinical study showed that LAC was used as adjuvant treatment for primary generalized uncontrollable tonic–clonic seizures and as monotherapy in partial‐onset seizures.^55^ To examine the antiseizure properties of PRP, both alone and in conjunction with LAC, we further lowered the dose in the current trial to 0.125 mg/kg. Remarkably, PRP at this dose of 0.125 mg/kg in combination with LAC 5 mg/kg effectively slowed down the kindling process; however, PRP at this dose of 0.125 mg/kg alone just slowed down the onset of kindling. Additionally, the administration of PRP 0.125 mg/kg in conjunction with LAC 5 mg/kg had positive results, as it prevented the animals from experiencing full‐bloom tonic–clonic convulsions.

The significant antiseizure effect of the PRP and LAC combination might be due to the synergistic effect of AMPA antagonism by PRP with LAC, which decreases neuronal firing by slow inactivation of the voltage‐gated sodium channel, which, in turn, reduces calcium influx and restores GABAergic function. These results support the superiority of multitargeted therapy over monotherapy. Our combination therapy is in line with other combination therapies done by M. Tariq et al. and W. Shakeel et al.^31^, ^56^

Literature states that the incidence of anxiety is greater in epileptic patients (about 45%).^57^ As a result, we used OFT, L/D, and EPM to analyze the anxiety behavior in every mouse associated with kindling process. The animals remained more time at the center of the OFT and light compartment of the L/D test and less time in the closed arms of the EPM, indicating that the monotherapy with PRP and LAC was successful. Mice receiving both medications at the same time had a notable improvement in the anxiolytic effects; however, the combined administration of the medicines resulted in a considerable drop in animal's exploratory behavior, as determined by the measures of locomotion identified in OFT. These results are corroborated by prior research, which demonstrated the anxiolytic action of PRP in a hole board experiment.^58^ Another preclinical study in mice supports the anxiolytic potential of LAC in marble burry test.^59^

Research shows that epilepsy is not the only condition that causes cognitive impairment; individuals with epilepsy may also have cognitive impairment from antiepileptic medications.^60^ In addition, mice were tested in MWM, PA, and NOR tasks to assess their memory and learning abilities. Compared to the healthy group, kindled mice exhibited poor cognitive skills, as they displayed thigmotaxic behavior, shorter latency, and a lower discriminating index. Nevertheless, mice receiving LAC and PRP monotherapy had a relative improvement in all the reported measures, but mice getting LAC and PRP dual treatment demonstrated much better neuroprotection and cognitive performance, exhibiting the protection of dual therapy against kindling‐associated memory impairment. Our findings are consistent with prior preclinical studies where LAC ameliorates the kindling‐associated impairment in memory and learning ability of rodents.^61^ Furthermore, our results about PRP are in line with the study where PRP reduced cognitive deficits in juvenile TLE mice via inhibiting expression of GluR1 and AP‐1.^62^

Depression is the most common psychiatric comorbidity present in epileptic patients, with prevalence of approximately 55%.^63^ In our study, mice's depression‐like behavior was evaluated using SPT and LH test. Compared to the kindled group, mice that received only LAC and PRP therapy showed fewer signs of depression. Mice receiving dual therapy of LAC and PRP exhibited superior neuroprotection as seen by their decreased depressed behavior, because these mice showed enhanced preference toward sweet water and shorter latencies in LH test. Short‐term prospective studies suggest that PRP may reduce or increase hostility and depressive‐like conduct in epileptic patients, with mild mood alterations tolerated in certain situations.^64^ In another rat model of absence epilepsy, long‐term administration of PRP at a dose range of 0.25–3 mg/kg significantly decreased comorbid depressive‐like behavior.^65^ According to a comprehensive analysis, adults with epilepsy that receive PRP as an add‐on medication have reduced symptoms of mood problems.^66^, ^67^

Disruption in normal balance of inhibition and excitation of neuronal activity leads toward hyperexcitability, which prominently accelerates the pathophysiological process, and leads to neuronal degeneration or death in epileptic brain.^68^ Excessive neuronal excitation increases the oxidative stress, which further aggravates the progression of epilepsy.^36^ In our study, biochemical analysis of the diseased mice brain homogenate showed enhanced MDA and AChE activity, whereas the activity of SOD, catalase, and GPx was reduced. These findings are in line with the previous studies that suggest corneal kindling induces oxidative stress in rodents.^69^, ^70^ However, the mice chronically treated with LAC and PRP alone showed less oxidative stress, whereas concomitant administration of LAC and PRP was more effective in reducing the epilepsy‐associated oxidative stress. Our results are consistent with those of previous studies where Lazzarotto et al. and Rocamora et al. reported the neuroprotective effect of LAC against oxidative stress associated with epilepsy.^66^, ^71^ Additionally, we observed that PRP administration reduces the oxidative stress in our study, and this outcome is supported by a previous study where PRP abates traumatic brain injury by reducing oxidative stress and neuroinflammation.^72^

## 5 CONCLUSION

The current study's findings indicated that monotherapy with LAC and PRP offered less protection against seizure development, but pharmacological intervention using combinatorial regime produced noticeable benefits in terms of seizure reduction during the kindling progression. Additionally, the animals given the combined treatment showed better behavioral gains, such as decreased anxiety and depression, and considerably improved cognitive capacities compared to the kindled mice. In addition, the combination‐treated group showed reduced levels of oxidative stress in isolated brains. Overall, coadministration of LAC and PRP might be working simultaneously to reduce kindling‐associated neuronal excitability through sodium channels and AMPA modulation.

## AUTHOR CONTRIBUTIONS

**Saba Tehreem:** Data curation; formal analysis; methodology; writing – original draft; writing – review and editing. **Azka Sabir:** Data curation; formal analysis; writing – original draft; writing – review and editing. **Maryam Farooq:** Formal analysis; software; visualization; writing – original draft; writing – review and editing. **Waseem Ashraf:** Conceptualization; investigation; methodology; project administration; supervision; writing – review and editing. **Faleh Alqahtani:** Formal analysis; funding acquisition; methodology; resources; validation; writing – review and editing. **Tanveer Ahmad:** Methodology; validation; writing – review and editing. **Imran Imran:** Conceptualization; funding acquisition; methodology; resources; supervision; validation; writing – original draft; writing – review and editing.

## FUNDING INFORMATION

The authors extended their appreciation to Distinguished Scientist Fellowship program at King Saud University, Riyadh, Saudi Arabia, for funding this work through research supporting project number (RSP2024R131).

## CONFLICT OF INTEREST STATEMENT

The authors declared no potential conflicts of interest.

## ETHICS STATEMENT

This research is approved by the Departmental Research Ethics Committee for the Department of Pharmacology, Faculty of Pharmacy, BZU, Multan and the approval number was 01‐PHL‐S22.

## Data Availability

The data that support the findings of this study are available from the corresponding author upon reasonable request.
